# Can Bacterial Endophytes Be Used as a Promising Bio-Inoculant for the Mitigation of Salinity Stress in Crop Plants?—A Global Meta-Analysis of the Last Decade (2011–2020)

**DOI:** 10.3390/microorganisms9091861

**Published:** 2021-09-02

**Authors:** Muhammad Aammar Tufail, Ana Bejarano, Awais Shakoor, Asif Naeem, Muhammad Saleem Arif, Afzal Ahmed Dar, Taimoor Hassan Farooq, Ilaria Pertot, Gerardo Puopolo

**Affiliations:** 1 Department of Civil, Environmental and Mechanical Engineering, University of Trento, Via Mesiano 77, 38123 Trento, Italy; muhammad.tufail@unitn.it; 2 Center Agriculture Food Environment (C3A), University of Trento, Via E. Mach 1, 38098 San Michele all’Adige, Italy; ilaria.pertot@unitn.it (I.P.); gerardo.puopolo@unitn.it (G.P.); 3 Department of Sustainable Agro-Ecosystems and Bioresources, Research and Innovation Centre, Fondazione Edmund Mach, Via E. Mach 1, 38098 San Michele all’Adige, Italy; 4 Department of Environment and Soil Sciences, University of Lleida, Avinguda Alcalde Rovira Roure 191, 25198 Lleida, Spain; awais.shakoor@udl.cat; 5 Institute of Plant Nutrition and Soil Science, Kiel University, Hermann-Rodewald-Strasse 2, 24118 Kiel, Germany; anaeem@plantnutrition.uni-kiel.de; 6 Department of Environmental Sciences & Engineering, Government College University Faisalabad, Faisalabad 38000, Pakistan; msarif@outlook.com; 7 School of Environmental Science and Engineering, Shaanxi University of Science and Technology, Xian 710000, China; afzaldar@sust.edu.cn; 8 Bangor College China, a Joint Unit of Bangor University and Central South University of Forestry and Technology, Changsha 410004, China; taimoorhassan2055@gmail.com

**Keywords:** plant growth-promoting endophytic bacteria, salinity stress, osmoregulation, antioxidant system, photosynthetic capacity, meta-analysis

## Abstract

Soil salinity is a major problem affecting crop production worldwide. Lately, there have been great research efforts in increasing the salt tolerance of plants through the inoculation of plant growth-promoting endophytic bacteria. However, their ability to promote plant growth under no-stress and salinity-stress conditions remains largely uncertain. Here, we carried out a global meta-analysis to quantify the plant growth-promoting effects (improvement of morphological attributes, photosynthetic capacity, antioxidative ability, and ion homeostasis) of endophytic bacteria in plants under no-stress and salinity-stress conditions. In addition, we elucidated the underlying mechanisms of growth promotion in salt-sensitive (SS) and salt-tolerant (ST) plants derived from the interaction with endophytic bacteria under no-stress and salinity-stress conditions. Specifically, this work encompassed 42 peer-reviewed articles, a total of 77 experiments, and 24 different bacterial genera. On average, endophytic bacterial inoculation increased morphological parameters. Moreover, the effect of endophytic bacteria on the total dry biomass, number of leaves, root length, shoot length, and germination rate was generally greater under salinity-stress conditions than no-stress conditions. On a physiological level, the relative better performance of the bacterial inoculants under the salinity-stress condition was associated with the increase in total chlorophyll and chlorophyll-b, as well as with the decrease of 1-aminocylopropane-1-carboxylate concentration. Moreover, under the salinity-stress condition, bacterial inoculation conferred a significantly higher increase in root K^+^ concentration and decrease in leaf Na^+^ concentration than under the no-stress condition. In SS plants, bacterial inoculation induced a higher increase in chlorophyll-b and superoxide dismutase activity, as well as a higher decrease in abscisic acid content, than in ST plants. Under salinity-stress, endophytic bacterial inoculation increased root K^+^ concentration in both SS and ST plants but decreased root Na^+^ concentration only in ST plants. Overall, this meta-analysis suggests that endophytic bacterial inoculation is beneficial under both no salinity-stress and salinity-stress conditions, but the magnitude of benefit is definitely higher under salinity-stress conditions and varies with the salt tolerance level of plants.

## 1. Introduction

Global land resources are adversely affected by a range of abiotic factors including soil salinity, which is one of the most relevant threats to agricultural production and food security [1]. It is estimated that there are about 1 billion hectares of salt-affected lands, with a definite upward tendency [2]. Soil salinity has already damaged around 20% of agricultural lands worldwide and this number is steadily increasing [3]. In the event of climate change, irrational irrigation methods, improper application of fertilizers, and inadequate drainage networks, this situation will get worst day by day. It is estimated that 50% of arable land will be under serious salinity risk by 2050 [4,5,6,7]. Soil salinity negatively affects many morphological and physical processes of plants including nutrients uptake, seed germination, and overall plant growth. Shortly after exposure to salinity, plants face an osmotic stress, which is followed by ion toxicity and nutrient imbalance. This condition, similar to water deficit, leads to the formation of hypertonic conditions outside the cell and impedes the plants to take up water. Subsequent ion toxicity is caused by the over accumulation of sodium (Na^+^) and chloride (Cl^−^) ions within the cells. Excessive amounts of Na^+^ and Cl^−^ damage plant cell walls, disturbs the osmotic balance, and modifies ion homeostasis within the cell, which ultimately induce changes in transpiration rate, translocations of nutrients, photosynthesis, and other metabolic processes [8]. In addition, soil salinity reduces soil microbial diversity/activity and the accumulation of organic matter. Thus, soils containing intermediate levels of salinity harbor higher amounts of bacteria than fungi, but at high levels of salinity, fungi growth is favored [9]. Saline soils are likely dominated by *Proteobacteria, Actinobacteria, Bacteroidetes*, and *Gemmatimonadetes*, but also by *Acidobacteria, Firmicutes, Nitrospirae*, and *Verrucomicrobia* [9] Yet, a relative abundance of *Bacteroidetes* and *Proteobacteria* has been positively correlated, while the abundance of *Acidobacteria* has been negatively correlated with high levels of salt [10].

To cope with salinity stress, plants have evolved different physiological mechanisms such as osmolyte aggregation, ion homeostasis, water absorption control, and antioxidants synthesis [11]. Regarding salinity stress tolerance, plants can be divided into salt sensitive (SS) and salt tolerant (ST) plants. A plant is considered SS when its growth is compromised even at low concentrations of NaCl (25 and 50 mM NaCl). Examples of very sensitive plants include chickpea (*Cicer arietinum* L.) and rice (*Oryza sativa* L.). In contrast, ST plants (e.g., *Salicornia europaea*) can survive and complete their life cycle in high salt concentrations (even higher than 200 mM NaCl) [12]. The main differences between SS and ST plants are based on their abilities to compartmentalize salt ions and synthesize organic solutes that contribute to the adjustment of the osmotic potential of the cytoplasm. In addition, depending on the mechanisms of adaptation to salinity stress, ST plants can be categorized as salt-excluding (intercept ions in roots and minimize the influx of Na^+^ to the shoot parts), salt-excreting (excrete absorbed salt to the outside), and salt-accumulating (accumulate salt ions in cytoplasmatic organelles known as vacuoles). It is undeniable that the responses of SS and ST plants to salt stress vary qualitatively and quantitatively. Not surprisingly, ST plants are prime candidates for exceedingly saline environments and thrive under conditions in which SS plants are either unproductive or inefficient. However, it is worth noting that both types of plants can undergo damage under salinity-stress conditions, especially at the early vegetative stage.

Moreover, plants establish interactions with a plethora of microorganisms that promote plant growth and mitigate plant stress [13]. Interestingly, the biodiversity of the plant microbiota varies with the level of salt tolerance of the plant [14]. Thus, ST plants commonly establish interactions with halotolerant plant growth-promoting bacteria, that is, bacteria that can survive in media containing up to 25% sodium chloride [15]. The most predominant halotolerant plant growth-promoting bacteria belong to *Halomonas, Bacillus, Streptomyces, Oceanobacillus*, and *Pseudomonas* [14]. The mechanisms of salinity resistance in halotolerant bacteria are mostly similar among different taxa. Thus, halotolerant bacteria overcome salinity via specific membrane or cell wall constructions, pumping ions out of the cell, accumulating compatible solutes, adapting proteins and enzymes to high concentrations of salt, augmenting cell’s energy capacity, or producing exopolysaccharides that limit the entry of salt into the cell [16]. Among all plant-associated bacteria, endophytes show to relive the impacts of salt stress in plants by inducing osmotic adjustment, detoxification, modulation of phytohormones, and acquisition of nutrients [17,18,19]. Endophytic bacteria with 1-aminocylopropane−1-carboxylate (ACC) deaminase and indole−3-acetic acid (IAA) production, nitrogen fixation, phosphate solubilization, and siderophore production traits have shown to promote the osmotic or ionic adaptation of host plants [20,21,22,23,24]. However, the exact endophytic bacterial-mediated mechanisms underlying salt stress alleviation remain largely unknown [25]. In this regard, integrating data across investigations may help to understand the extent to which bacterial endophytes mitigate salt stress and ultimately contribute to the broader use of endophytic bacteria in sustainable agriculture.

A meta-analysis is a tool that synthesizes knowledge using a specific methodological procedure for data aggregation and analysis from various individual scientific studies [26]. It is particularly useful for answering study questions of great versatility and uncovering emergent properties within individual studies that would otherwise go undetected. The power of a meta-analysis becomes obvious when the outcomes of particular experiments vary in various experimental conditions. Recently, a meta-analysis was conducted to compare the overall effects of organic amendments on nitrous oxide (N_2_O) emission from agricultural soils and to examine which soil physicochemical properties and agricultural management practices are the main driving factors for N_2_O emission. This meta-analysis showed that, overall, biochar amendment mitigates N_2_O emission, while animal manure significantly increases it. Moreover, it revealed that the level of emitted N_2_O varies with soil texture, pH, and the C:N ratio [27]. Another recent meta-analysis was carried out to determine the potential of biochar for the bioremediation of heavy metals in contaminated soil and plant environments. Authors demonstrated that the immobilization of heavy metals can be a function of physicochemical properties of biochar and evidenced that the potential of biochar to relegate the metal toxicity is greatly influenced by edaphic factors and experimental methods [28].

To date, a few meta-analyses have reported the effect of the inoculation of plant growth-promoting rhizobacteria to improve the abiotic stress tolerance of plants [7,29,30]. For instance, the overall effect of endophytic bacterial inoculation to improve plant heavy metal tolerance has been recently published by Franco-Franklin and his co-workers [31]. Nevertheless, to the best of our knowledge, only Rho et al. [32] have attempted to measure the overall effect of bacterial and fungal endophytes on plants subjected to different abiotic stresses such as salinity, drought, and nitrogen stress in a meta-analysis. Moreover, so far there are no meta-analyses addressing the effects of endophytic bacteria on SS or ST plants under salinity-stress conditions.

Here, we combined data from 42 articles and performed a meta-analysis for assessing the efficacy of endophytic bacterial inoculation in the mitigation of salinity stress in plants. Moreover, we classified the host plants into SS and ST groups and compared the effects of bacterial endophyte inoculation on two types of host plants. Specifically, we hypothesized that (i) endophytic plant growth-promoting bacteria are more effective under salinity-stress and (ii) salinity stress mitigation conferred by endophytic bacteria varies across SS and ST plants.

## 2. Materials and Methods

### 2.1. Database Search and Selection Criteria

Metadata was obtained following PRISMA reporting guidelines [33,34]. A literature search was conducted in December 2020 using SCOPUS^®^ (http://www.scopus.com (accessed on 31 December 2020)) and Web of Science^®^ (https://webofknowledge.com/ (accessed on 31 December 2020)) databases. Only articles published in scientific journals in English were retrieved using the following combination of keywords: “plant growth promot*” AND “endophyt*” AND “bacteria*” AND (“salinity” OR “salt”) AND “stress”. The Boolean truncation (‘*’) character was included to ensure the variations of the words, such as promoting or promotion, endophyte or endophytic, and bacteria or bacterial. The logical operator AND was used to refine articles that contained words written on both sides of the operator. The decision regarding the inclusion or exclusion of an article in the study was made with mutual discussion between the authors.

### 2.2. Study Selection

Research Metadata search from both databases yielded 227 articles, of which 150 remained after duplicate removal. To eliminate publication bias, the following eligibility criteria were predefined:

1. The study should contain at least one bacterial endophyte irrespective of the plant colonization rate. Bacterial endophytes should not necessarily be halotolerant.
2. Bacterial inoculum should not include additives such as amino acids, humic acids, protein hydrolysates, etc.
3. Both bacterial-inoculated and non-inoculated plants must have been evaluated under salinity-stress and no-stress conditions. If several levels of salinity stress are investigated in a study, the highest level shall be selected for this analysis.
4. Either the parameter of biomass (yield and weight) or plant height must have been reported in the study.
5. The results should have reported the means, standard deviations/errors, sample size, and other relevant statistical information to calculate the effect size.

The studies not fulfilling the above criteria were excluded from the analysis. If any of the traits were measured over time, only the last time point was considered. From the identified 150 articles, only 42 met our selection criteria and thus were moved forward to the analysis (Figure S1).

### 2.3. Data Extraction

Treatment means, standard deviations, and sample size (number of replications (*n*)) were extracted from each study. If the standard error (SE) was given in a study, it was converted into the standard deviation (SD) using the following equation $:\;SD=SE\;\sqrt{n}$. Data given in the form of graphs were digitized using WebPlotDigitizer [35]. Considering multiple experiments from one study do not increase the dependence of the meta-analysis on that study [36], different treatments or host/endophyte variants from the same article were regarded as independent experiments. This technique increases the power of the meta-analysis [37] and has been used in several meta-analyses [38,39,40].

Parameters related to plant morphology, plant physiology, enzymes and antioxidants, and ion homeostasis were collected from each study. To maintain the heterogeneity in each observation, parameters found in less than five data units were excluded from the study.

### 2.4. Meta-Analysis

To estimate the effect sizes of bacterial endophytes under no-stress and salinity-stress conditions, log response ratios (ln*RR*) were calculated as the matrices of effect sizes using the following formula: $\ln RR=\ln\left(V_{i}/V_{c}\right)$, where *V_i_* is the mean of the inoculated treatments and *V_c_* is the mean of the non-inoculated treatments [41]. Calculating ln*RR* as an effect size metric is appropriate because the log transformation of the parameter(s) reported in different units among studies maintains symmetry within the analysis [42]. Furthermore, percent change (%Δ) can be calculated easily from ln*RR* as follows: $\%\Delta =\left(e^{\ln RR}-1\right)\times 100$. Pooled variances were calculated using the “escalc” function in the “metafor” (version 2.4-0) package [43] of the R environment, version R-4.0.4 (https://r-project.org/ (accessed on 31 December 2020)).

A heterogeneity test was performed before constructing the meta-analysis model to determine the choice of either a fixed or random/mixed effect model. According to Cochran’s Q test, heterogeneity (Q) of the full dataset (*n* = 1214 observations) was highly significant (Cochran’s Q = 164278, df = 1213, *p* < 0.001) [44].

The data synthesis produced by the random/mixed effects meta-analysis was balanced based on the weight of each study to maintain their equal contribution to the results produced by the meta-analysis. In this study, the inverse variance method was used to assign the weights using meta [45] and metafor [43] packages in R. Estimated pooled effect sizes produced by the meta-analysis with their 95% confidence intervals (95% CI) were presented in forest plots created with ggplot [46] in R. The effect of inoculation with bacterial endophytes was considered significant if 95% CIs did not coincide with the zero line. Overlaps on the zero line mean that there was no significant effect of inoculation and it is denoted by ‘ns’ [47]. A positive value indicates an increase and a negative value indicates a decrease in the effect size of plants inoculated with endophytic bacteria, which are denoted by percent change (±%).

The overall summary effects of each condition (non-stress and salinity stress) were additionally grouped into SS and ST plants. SS plants compared the effects on plants that are sensitive even at low concentrations of NaCl (25 and 50 mM NaCl), while ST plants compared the effects on plants that could resist up to 200 mM NaCl [48].

## 3. Results

### 3.1. Metadata

**Figure 1:**
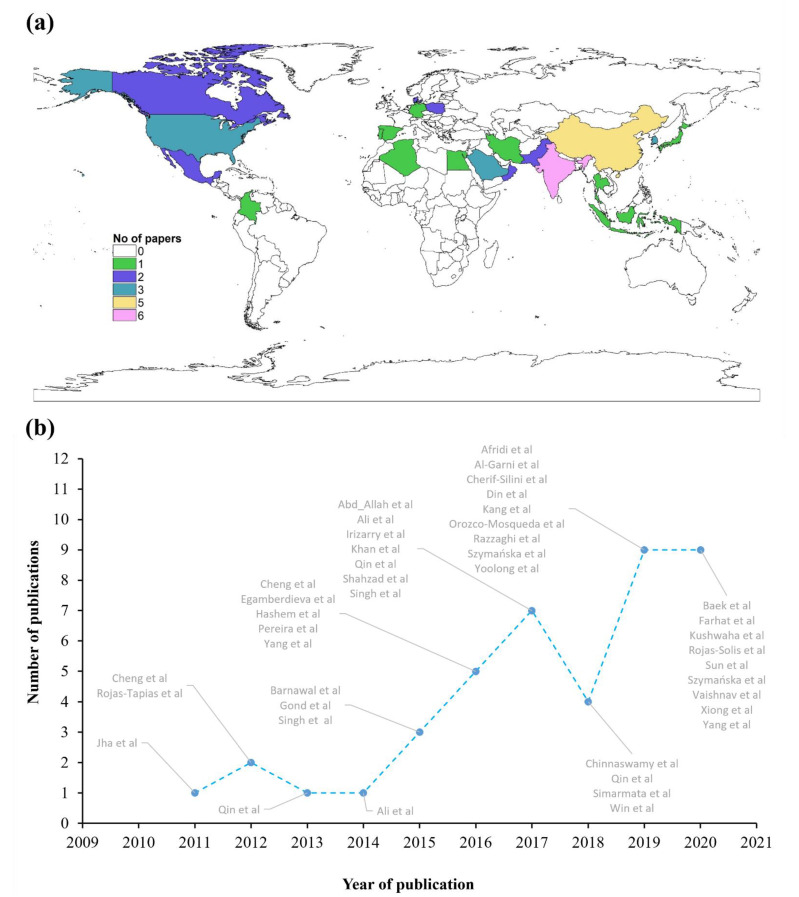
(**a**) Location of the experiments obtained from the selected studies (42) used in this meta-analysis (https://www.r-spatial.org/r/2018/10/25/ggplot2-sf.html (accessed on 31 December 2020)) and (**b**) the accumulated number of publications reported within the last 10 years (2011–2020) used in this meta-analysis. Data labels on each scatter point show the author names in that year.

Metadata was extracted from 42 peer-reviewed articles published in 21 different countries between 2011 and 2020 (Figure 1a,b). A total of 1214 observations (k) were obtained from a sum of 77 experiments. For each study, we used uniform selection criteria, which involved endophytic bacterial inoculants and their usefulness for crop plants in both no-stress and salinity-stress conditions. Seed inoculation was used in 60% (k = 632) of observations, while seedling and soil inoculation methods were used in 26% (k = 316) and 14% (k = 266) of observations, respectively (Figure S2a). The majority of the experiments (64%) were conducted in pots, followed by in-vitro (27%), hydroponic (6%), and growthroom (3%) (Figure S2b). In total, 24 bacterial genera, including 15 gram-negative and 9 Gram-positive, were identified from the extracted metadata (Figure S2c). Among Gram-negative bacteria, *Pseudomonas* and *Pantoea* were the most represented genera, whereas *Bacillus* was the most represented genus in the case of Gram-positive bacteria. Many of those, but not all, were considered halotolerant bacteria.

### 3.2. Effects of Endophytic Bacterial Inoculation on the Plant Morphological and Physiological Parameters

**Figure 2:**
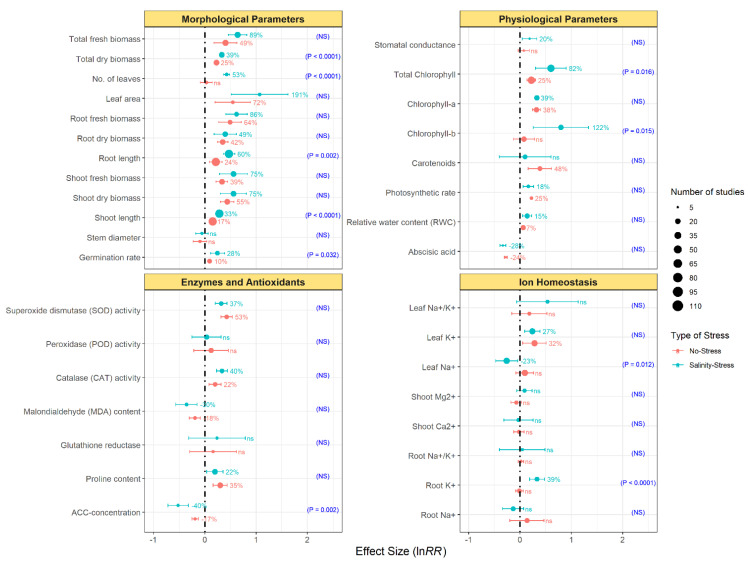
Effect of endophytic bacterial inoculation on morphological parameters, physiological parameters, enzymes and antioxidants, and ion homeostasis under no-stress and salinity-stress conditions. Error bars represent 95% confidence intervals (CIs). The inoculation effects were considered significant if the 95% CIs did not overlap with the zero line. The size of the scatter point shows the number of experimental observations. *p*-values and ‘NS’ in parenthesis show the significant and non-significant differences, respectively, between growth conditions.

In general, endophytic inoculation significantly enhanced the plant morphological-related parameters (i.e., total dry and fresh biomass, number of leaves, leaf area, root dry and fresh biomass, shoot dry and fresh biomass, root and shoot length, and germination rate) (Figure 2). This positive effect occurred in both the no-stress and salinity-stress conditions. Yet, the effect size was larger when endophytic inoculation was carried out under salinity stress. In fact, endophytization increased the magnitude of the plant growth promotion by 28–191% in salinity-stressed plants, while in no-stressed plants, this increase ranged from 10% to 72%. Moreover, the effect sizes on the dry biomass, number of leaves, root length, shoot length, and germination rate were significantly higher under salinity-stress conditions (Figure 2).

The effect of endophytic bacterial inoculation was also significantly higher for most of the plant physiological attributes. Thus, endophytic bacterial inoculation increased total chlorophyll, chlorophyl a, photosynthetic rate, and the relative water content (RWC) of plants across all conditions (Figure 2). Additionally, the inoculation of plants with endophytic bacteria resulted in a decrease of the leaf abscisic acid content. Endophytic bacterial inoculation generally led to greater effect sizes of physiological parameters in stressed plants than in non-stressed controls and solely the carotenoids and photosynthetic rate followed the opposite pattern, with endophytic inoculation accounting for the greater effects size under the no-stress conditions. Nevertheless, only the effects on total chlorophyll and chlorophyll b content differed between non-stressed and salinity-stressed plants (Figure 2).

### 3.3. Effect of Endophytic Bacterial Inoculation on Plant Antioxidant Enzymes and Ionic Homeostasis

Endophytic bacterial inoculation led to a significant increase in antioxidant activity (e.g., superoxide dismutase (SOD) and catalase (CAT)) and proline content both under no-stress and salinity-stress conditions. Moreover, endophytic inoculation greatly decreased malondialdehyde (MDA) and ACC-concentration content, especially under salinity stress. In contrast, peroxidase (POD) activity and glutathione reductase were not affected by the endophytic bacterial inoculation, irrespective of the stress (Figure 2). As for ion homeostasis, endophytic bacterial inoculation increased K^+^ concentration in leaves in both non-stressed and salinity-stressed plants in a similar manner (Figure 2). Conversely, the content of K^+^ in roots was only increased in stressed plants (Figure 2; *p* < 0.0001). Similarly, endophytic bacterial inoculation decreased the content of leaf Na^+^ (by a 23%) only in stressed plants.

### 3.4. Comparative Effects of Endophytic Bacterial Inoculation on the Growth of Salt-Sensitive and Salt-Tolerant Plants

**Figure 3:**
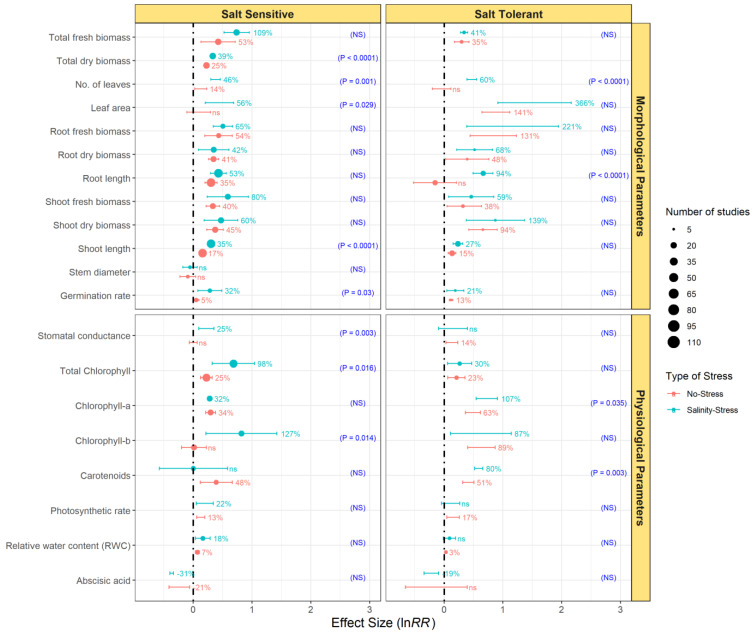
Effect of endophytic bacterial inoculation on morphological parameters and physiological parameters of salt-sensitive and salt-tolerant plants under no-stress and salinity-stress conditions. Error bars represent 95% confidence intervals (CIs). The inoculation effects were considered significant if the 95% CIs did not overlap with the zero line. The size of the scatter point shows the number of experimental observations. *p*-values and ‘NS’ in parenthesis show the significant and non-significant differences, respectively, between growth conditions.

Concerning SS plants, endophytic bacterial inoculation increased the total fresh and dry biomass, root dry and fresh biomass, number of leaves, leaf area, shoot fresh and dry biomass, root and shoot length, and germination rate (Figure 3). Total fresh biomass and shoot fresh biomass were the most responsive parameters to endophytic bacterial inoculation, followed by root fresh biomass. Interestingly, the effect size of the total dry biomass, number of leaves, leaf area, shoot length, and germination rate in SS plants was significantly larger under salinity-stress than no-stress conditions (Figure 3; *p* < 0.05). Similarly, for ST plants, endophytic inoculation had a general positive effect on plant morphological parameters. In terms of stress conditions, endophytic inoculation significantly increased the number of leaves and root length under salinity stress conditions (Figure 3; *p* < 0.0001).

Endophytic bacterial inoculation also enhanced physiological parameters in SS and ST plants, especially under salinity-stress conditions (Figure 3). Thus, endophytization increased the stomatal conductance and content of total chlorophyll, chlorophyll-a, chlorophyll-b, carotenoids, photosynthetic rate, and RWC in both SS and ST plants. Under salinity stress conditions, endophytic bacterial inoculation significantly increased the total chlorophyll and chlorophyll-b in SS plants, and chlorophyll-a and carotenoids in ST plants (Figure 3). Moreover, in SS plants, there was a significantly higher endophyte effect on the stomatal conductance (*p* = 0.003) under salinity-stress than no-stressed controls. Inoculation of plants with endophytic bacteria decreased the abscisic acid content in SS plants grown under both no-stress and salinity-stress conditions by 21% and 31%, respectively. As for ST plants, endophytic bacteria decreased the abscisic acid content only in plants subjected to salinity stress. However, the effect of endophytization on the abscisic acid content did not differ across conditions either in SS or ST plants.

**Figure 4:**
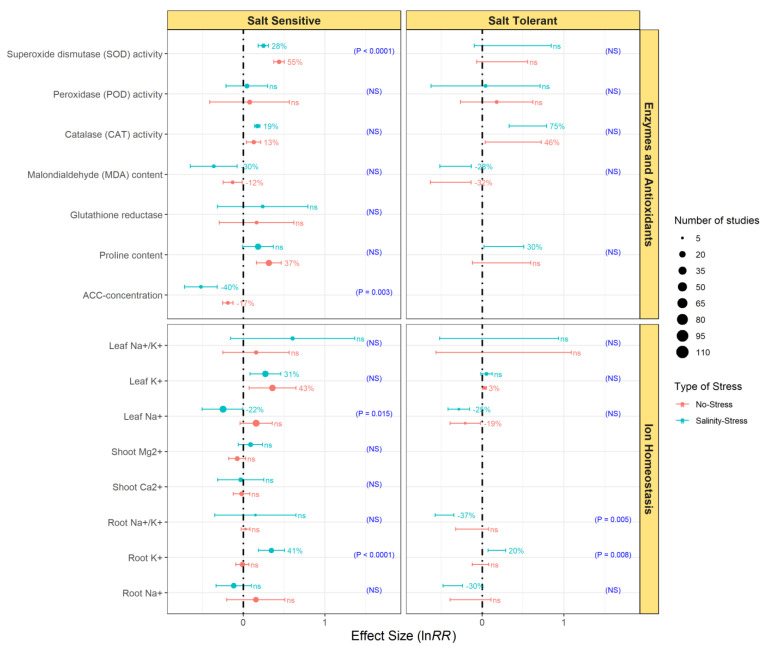
Effect of endophytic bacterial inoculation on enzymes and antioxidants, as well as on the ion homeostasis of salt-sensitive and salt-tolerant plants under no-stress and salinity-stress treatments. Error bars represent 95% confidence intervals (CIs). The inoculation effects were considered significant if the 95% CIs did not overlap with the zero line. The size of the scatter point shows the number of experimental observations. *p*-values and ‘NS’ in parenthesis show the significant and non-significant differences, respectively, between growth conditions.

Overall, the effects of endophytic bacterial inoculation on enzymes and antioxidants’ activity in SS plants were statistically significant across all growth conditions (Figure 4). Endophytic bacteria significantly enhanced CAT and SOD activity, while the MDA and ACC-concentration were significantly decreased (Figure 4). Moreover, the effects of endophytization on SOD activity and ACC concentration differed between the non-stressed and salinity-stressed plants (Figure 4). Inoculation of ST plants with endophytic bacteria uniquely led to a significant decrease of MDA contents in both no-stress and salinity-stress conditions. However, the effect size of inoculation did not differ between those conditions.

On average, endophytic bacterial inoculation significantly increased levels of K^+^ in both leaf and root tissues, and decreased leaf Na^+^ in SS plants subjected to salinity-stress (Figure 4). Similarly, under salinity stress, endophytic bacterial inoculation significantly increased K^+^, while decreasing Na^+^ and Na^+^/K^+^ levels in roots of ST plants (Figure 4). Endophytic bacterial inoculation also decreased the leaf Na^+^ content in salt-tolerant plants under both no-stress and salinity-stress conditions, although the effect of inoculation did not differ between conditions.

## 4. Discussion

Over the last 50 years, agricultural intensification has resulted in higher crop yields, but salinity stress is severely limiting the growth and yield potential of crops worldwide [49,50], putting food security at risk. The breeding and production of transgenic plants are considered practical approaches to enhance the salt tolerance of plants [51,52]; however, they have often failed to efficiently alleviate the situation. Our meta-analysis on the subject matter shows that salinity stress has garnered a great deal of attention from the scientific community in the last two decades. Indeed, a constant increase of scientific publications has been observed over this period. More importantly, it gathers valuable findings from 77 experiments evaluating the effect of endophytic bacteria on plant growth under diverse environmental conditions.

The use of Gram-positive bacteria was common among the studies selected for this meta-analysis. This is especially relevant as the impact of Gram-positive bacteria on plant growth is less documented compared to the impact of Gram-negative bacteria [53]. Importantly, many Gram-positive bacteria are spore-forming, produce numerous bioactive compounds and secondary metabolites, and have specialized lifestyles that could be advantageous for agricultural applications [53]. In contrast, Gram-negative bacteria do not form spores and are well studied mainly due to the symbioses between Gram-negative rhizobia and legume crops [54]. Our meta-analysis revealed that seed inoculation was widely used as a method for inoculation of endophytic bacteria. This method of inoculation is a relatively efficient approach for the introduction of bacteria into the soil [55], especially in the case of salinity stress. Notably, our analysis showed that most of the experiments with endophytic bacteria were performed in pot experiments. This information highlights that a successful strategy for the application of products for the field scale has yet to be realized, despite evidence that endophytic bacteria might improve crop production.

The magnitude of plant adaptations to salinity stress is typically assessed by the gains in plant biomasses [8]. Indeed, our meta-analysis showed that endophytic bacterial inoculation had a positive impact on biomass production, which is in accordance with previous findings [32]. This positive effect was even more noticeable when plants were grown under salinity stress conditions. A possible explanation for this might be found in the ability of endophytic bacteria to stimulate greater changes in physiological activity, antioxidant activity, photosynthesis, osmoregulation, and the ion homeostasis of plants grown under salinity stress conditions [56,57,58]. Reducing the leaf area is a common reaction of plants to salt stress. Indeed, the first reaction of SS plants to salt stress is to reduce the leaf development and number of leaves. This action may be interpreted as an avoidance mechanism to minimize water loss through transpiration, as it facilitates the retention of deleterious ions in the root system, minimizing their build-up in plants’ leaves [59]. In response to salinity, plants also loose leaf turgor and lower photosynthetic rates, which ultimately results in a decrease in the total leaf area and therefore biomass [59]. The present meta-analysis evidenced that the inoculation of plants with endophytic bacteria leads to an increase in the number of leaves and leaf area, which might be due to the positive regulation of phytohormones or enzymes and antioxidants activity. In fact, the inoculation of tomato plants with *Pseudomonas* spp. enhanced the leaf area under salinity stress, which has been related to the production of ACC-deaminase by the bacterium [60].

Chlorophyll and carotenoids are important pigments of the photosynthesis that convert solar energy into the rich organic molecules needed for the growth of plants [61]. Sugars and carbohydrates play critical roles in signaling and defending stressed plants, as they serve as the primary structural framework and energy supply for biomass processing and maintenance [62]. This meta-analysis showed that bacterial inoculation improved chlorophyll and carotenoid contents under both no-stress and salinity-stress conditions. Moreover, our meta-analysis evidenced that under salinity stress conditions, endophytic bacterial inoculation increases chlorophyll contents in a greater extent compared to no-stress conditions, in line with recent findings [23,63]. This suggests that improvements in biomass and other morphological-related parameters of salinity-stressed plants might be attributed to an increased photosynthetic activity stimulated by the application of endophytic bacteria. In addition, the inoculation of plants with endophytic bacteria decreased the leaf abscisic acid content under salinity-stress conditions. This hormone is responsible for stomata closure [64] and the accumulation of osmotically active substances [65]. Typically, stressed plants accumulate high levels abscisic acid content. However, the effect of an increased abscisic acid content can be contradictory as high levels of abscisic acid may also have negative impacts on plants. Previous research showed that inoculation of wheat with the plant growth-promoting bacteria *Bacillus subtilis* and *Pseudomonas mandelii* led to a decrease in the level of the abscisic acid in shoots [66]. In line with our findings, this decrease came alongside an increase in the leaf area and chlorophyll levels, which suggests that a bacterial-induced decrease in leaf abscisic acid is likely to be implicated in maintaining the level of photosynthesis of inoculated plants. Interestingly, endophytic bacterial inoculation significantly increased the activity of the reactive oxygen species (ROS)-scavenging enzymes CAT and SOD and decreased MDA content, thereby contributing to preventing tissues from oxidative damages [67]. An expression analysis of stress-responsive genes revealed that the higher activity of the ROS-scavenging enzymes, SOD, CAT, ascorbate peroxidase, dehydroascorbate reductase, and glutathione reductase came alongside the up-regulation of expression levels of the corresponding genes in *Solanum tuberosum* inoculated with *Bacillus firmus* and *Bacillus pumilus* [68]. In general, oxidative stress caused by salinity decreases photosynthesis by modifying photosynthetic pigments and reducing the photosynthetic rate [69]. Thus, improved photosynthesis in inoculated plants may be also linked to an improved production of antioxidants within plants that counteracted the destruction of chlorophylls and carotenoids caused by ROS [70,71]. In support of this hypothesis, *Bacillus* also improved the photosynthetic performance in *Solanum tuberosum* subjected to salt stress [68].

High levels of salinity lower the osmotic potential of soil water, leading to a reduction in water uptake by plant roots [11,72]. In this context, plant osmoregulation becomes an essential mechanism to overcome plant osmotic stress triggered by high salt concentrations [73]. However, it is worth noting that plants expend the bulk of their energy to accumulate and synthesize osmolytes during osmoregulation, with a negative effect on the plant biomass [74,75]. Recently, it has been reported that osmoregulation can be assisted by endophytic bacteria [71]. The significant increase in proline concentration in plants inoculated with endophytic bacteria might be one of the possible mechanisms that plants implement to overcome the osmotic stresses. Indeed, proline has been proved to be involved in the plant osmoregulation [76,77], stabilization of cellular structure, and reduction of damage in the photosynthetic apparatus [78]. As an example, the *Enterobacter* species up-regulated the expression of salt stress-responsive genes related to proline biosynthesis in *Arabidopsis thaliana* [79].

Ethylene is a gaseous plant hormone and is required by plants in very low quantities (commonly less than 1.0 μL L^–1^) for growth and development. Indeed, low concentrations of ethylene can trigger the germination of seeds and development of roots, leaves, and flower primordium, as well as the elongation of roots [80,81]. Under stress conditions, the level of ethylene in plants increases above the critical threshold, typically inhibiting plant growth [82]. As a precursor of ethylene, ACC is converted into ethylene by an ACC oxidase [83]. Endophytic bacteria can influence the production of ethylene in plants through the enzymatic action of ACC deaminase. This enzyme transforms ACC into ammonia and α-ketobutyrate in plants, lowering the levels of ACC within plants. As a result, ACC deaminase reduces the levels of ethylene that are detrimental for plant growth under environmental stresses [84]. The ACC deaminase containing endophytic bacterium species *Enterobacter* P23 has mitigated the effects of salt stress (0 and 150 mM NaCl) and promoted the growth of rice plants by reducing ethylene levels in plants [85]. Our meta-analysis suggested that the inoculation with endophytic bacteria improves plant growth under salinity stress by lowering the ACC concentration in plant tissues, utilizing the mechanistic action of ACC-deaminase, which in turn reduces ethylene toxicity in plants.

In events of high salinity, Na^+^ interferes competitively with a range of core physiological functions that depend on K^+^ [86]. Hence, the modulation of the interaction between Na^+^ and K^+^ is widely accepted as a measure that plants may implement to tolerate salt stress [86,87,88]. Our meta-analysis suggests that endophytic bacteria help plants to maintain ion homeostasis by regulating the accumulation of Na^+^ and K^+^. The increase in the level of K^+^ in roots and leaves and the decrease of the level of Na^+^ in leaves upon inoculation indicate that they might be key mechanisms by which bacterial endophytes can ameliorate salinity stress. In fact, it has been reported that *B. subtilis* down-regulated the expression of the high-affinity K^+^ transporter *HKT1* in the roots of plants grown under salinity stress, ultimately reducing the uptake of Na^+^. Intriguingly, at the same time it up-regulated *HKT1* in the shoots, this manner facilitating shoot-to-root Na^+^ recirculation [89]. Another transcriptome analysis revealed that the halotolerant plant growth-promoting bacteria *Dietzia natronolimmanea* enhanced the expression of genes related to ion transporters, SOS pathway, and antioxidants in wheat, thereby protecting plants from salinity stress [90]. Similarly, Liu et al. [91] showed that that *Bacillus amyloliquefaciens* up-regulated genes related to Na^+^ translocation, photosynthesis, auxin, ROS-scavenging, and osmoprotectants, as well as ethylene and jasmonic acid signaling under salt stress conditions in *Arabidopsis thaliana*. In general terms, the efficacy of a plant to regulate Na^+^ absorption, distribution, and compartmentalization depends on its salt resistance [48]. However, the growth-promoting effect of endophytic bacterial inoculation was not limited to only ST plants. SS plants also exhibited substantial improvements in plant morphological parameters, photosynthesis, antioxidants production, and ion homeostasis upon endophytic bacterial inoculation. This positive effect might be related to the higher effect on the stomatal conductance and the content of total chlorophyll and chlorophyll-b, SOD activity, and ACC concentration showed by endophytic bacteria in SS plants than in ST plants. Under salinity stress, endophytic bacteria increased root K^+^ concentration in both SS and ST plants but decreased root Na^+^ concentration only in ST plants. ST plants achieved salt tolerance either by excluding most of the Na^+^ and Cl^–^ in the soil solution or by accumulating salt ions in the roots and root-stem junctions [92]. This indicates that Na^+^ exclusion might be an inherited plant trait and endophytic bacteria failed to induce it SS plant species. Thus, it might be conceivable that endophytic bacteria would induce salt tolerance in SS plants only through the increase of K^+^ uptake by roots. However, it is not possible to formulate a definite conclusion as only a few studies investigated the effect of endophytic bacterial inoculation in ST plants subjected to salinity stress.

In brief, this meta-analysis supports the value of endophytic bacteria in the alleviation of salinity stress in plants. However, a microbial strain performing well in vitro may perform badly under greenhouse or field conditions [93]. Field-introduced microbes must overcome many hurdles before reaching the desired plant and exerting the desired plant growth-promoting effects. They must survive under the pressure of abiotic stressors, establish interactions with the indigenous microbiota, and colonize the plant [94]. The heterogeneity and limited reliability of bacteria-based biofertilizers under non-controlled conditions can be attributed to edaphic and environmental circumstances [95]. Therefore, future work should focus on finding a way to maximize the use of bacterial endophytes in the field. For instance, by examining the ability of each microorganism to adapt to extreme conditions through the manipulation of its growth conditions and by developing protective formulations for field application [96].

## 5. Conclusions

This meta-analysis, including 42 articles, 77 experimental units, and 1214 observations, and spanning over 10 years (2011–2020), suggests that endophytic bacteria enhance plant growth by improving physiological parameters (e.g., leaf area, chlorophyll content, and RWC) and antioxidant enzyme activity (SOD and CAT), decreasing MDA concentrations, and enhancing K^+^ acquisition and Na^+^ exclusion. Moreover, our analysis suggests that endophytic bacterial inoculation is beneficial under both no-stress and salinity-stress conditions, but the magnitude of benefit is definitely higher under salinity stress conditions. Inoculation of endophytic bacteria had a positive effect in SS and ST plants. However, SS plants failed to exclude Na^+^ even with the inoculation of endophytic bacteria and the increase in K^+^ uptake remains as the main mechanism underlying bacterial-induced salt tolerance. Ultimately, this meta-analysis establishes that the inoculation of plant growth-promoting bacterial endophytes is an effective tool for improving plant growth under salinity and no-stress conditions.

## Data Availability

The data presented in this study are openly available online at https://www.mdpi.com/article/10.3390/microorganisms9091861/s1. Selected publication references and Meta-Data.

## Supplementary Materials

The following are available online at https://www.mdpi.com/article/10.3390/microorganisms9091861/s1, Figure S1: Preferred reporting items for systematic reviews and meta-analyses (PRISMA) flow diagram for the meta-analysis (Moher et al., 2009, https://doi.org/10.1371/journal.pmed.1000097 (accessed on 21 July 2009)), Figure S2: General information about the 1214 observations and 77 experiments obtained from 42 studies used in this meta-analysis, (a)- Inoculation method, (b)- Experimental conditions, and (c)- Genera of bacterial endophytes.
