# Supplementary material for: Can Bacterial Endophytes Be Used as a Promising Bio-Inoculant for the Mitigation of Salinity Stress in Crop Plants?—A Global Meta-Analysis of the Last Decade (2011–2020)
Source: Microorganisms. 2021 Sep 2;9(9):1861. doi: 10.3390/microorganisms9091861 (PMC8467090; doi:10.3390/microorganisms9091861)
Supplement: Supplementary file 1 [file microorganisms-09-01861-s001.zip › Selected publications reference.pdf]

microorganisms

MDPI

Article

Can bacterial endophytes be a promising bio-inoculant for crop plants to combat salinity stress-A global meta-analysis

Muhammad Aammar Tufail[1,2,3], Ana Bejarano[2,3], Awais Shakoor[4], Asif Naeem[5], Muhammad Saleem Arif[6], Afzal Ahmed Dar[7], Taimoor Hassan Farooq[8], Ilaria Pertot[2,3] and Gerardo Puopolo[2,3]

[1] Department of Civil, Environmental and Mechanical Engineering, University of Trento, via Mesiano 77, 38123, Trento, Italy; muhammad.tufail@unitn.it (M.A.T.)
[2] Department of Sustainable Agro-ecosystems and Bioresources, Research and Innovation Centre, Fondazione Edmund Mach, Via E. Mach 1, 38010, San Michele all'Adige, Italy
[3] Center Agriculture Food Environment (C3A), University of Trento, via E. Mach 1, 38010, San Michele all'Adige, Italy; ana.bejaranoramos@unitn.it (A.B.), gerardo.puopolo@unitn.it (G.P.)
[4] Department of Environment and Soil Sciences, University of Lleida, Avinguda Alcalde Rovira Roure 191, 25198, Lleida, Spain; awais.shakoor@udl.cat (A.S.)
[5] Institute of Plant Nutrition and Soil Science, Kiel University, Hermann-Rodewald-Strasse 2, 24118, Kiel, Germany; anaeem@plantnutrition.uni-kiel.de (A.N.)
[6] Department of Environmental Sciences & Engineering, Government College University Faisalabad, Faisalabad, 38000, Pakistan; msarif@outlook.com (M.S.A.)
[7] School of Environmental Science and Engineering, Shaanxi University of Science and Technology, 710000, Xian, China; afzaldar@sust.edu.cn (A.A.D)
[8] College of Life Science and Technology, Central South University of Forestry and Technology, Changsha, 410004, Hunan, China; taimoorhassan2055@gmail.com (T.H.F)

* Correspondence: e-mail@e-mail.com; Tel.: (optional; include country code; if there are multiple corresponding authors, add author initials)

Papers selected in this meta-analysis: Based on our selection criteria and discussion among authors, following peer-reviewed articles were selected in this meta-analysis [1–42].

1. Yang, A.; Akhtar, S.S.; Fu, Q.; Naveed, M.; Iqbal, S.; Roitsch, T.; Jacobsen, S.E. *Burkholderia phytofirmans* PsJN stimulate growth and yield of quinoa under salinity stress. *Plants* **2020**, *9*, doi:https://doi.org/10.3390/plants9060672.
2. Xiong, Y.-W.; Li, X.-W.; Wang, T.-T.; Gong, Y.; Zhang, C.-M.; Xing, K.; Qin, S. Root exudates-driven rhizosphere recruitment of the plant growth-promoting rhizobacterium *Bacillus flexus* KLBMP 4941 and its growth-promoting effect on the coastal halophyte *Limonium sinense* under salt stress. *Ecotoxicol. Environ. Saf.* **2020**, *194*, 110374, doi:https://doi.org/10.1016/j.ecoenv.2020.110374.
3. Vaishnav, A.; Singh, J.; Singh, P.; Rajput, R.S.; Singh, H.B.; Sarma, B.K. *Sphingobacterium sp.* BHU-AV3 induces salt tolerance in tomato by enhancing antioxidant activities and energy metabolism. *Frontiers in Microbiology* **2020**, *11*, 13, doi:https://doi.org/10.3389/fmicb.2020.00443.
4. Szymańska, S.; Tyburski, J.; Piernik, A.; Sikora, M.; Mazur, J.; Katarzyna, H. Raising beet tolerance to salinity through bioaugmentation with halotolerant endophytes. *Agronomy* **2020**, *10*, 1571, doi:https://doi.org/10.3390/agronomy10101571.
5. Sun, L.; Lei, P.; Wang, Q.; Ma, J.; Zhan, Y.; Jiang, K.; Xu, Z.; Xu, H. The endophyte *Pantoea alhagi* NX-11 alleviates salt stress damage to rice seedlings by secreting exopolysaccharides. *Frontiers in Microbiology* **2020**, *10*, 3112, doi:https://doi.org/10.3389/fmicb.2019.03112.

6. Rojas-Solis, D.; Vences-Guzmán, M.Á.; Sohlenkamp, C.; Santoyo, G. Antifungal and plant growth–promoting *Bacillus* under saline stress modify their membrane composition. *J. Soil Sci Plant Nutri.* **2020**, *20*, 1549-1559, doi:https://doi.org/10.1007/s42729-020-00246-6.
7. Kushwaha, P.; Kashyap, P.L.; Kuppusamy, P.; Srivastava, A.K.; Tiwari, R.K. Functional characterization of endophytic bacilli from pearl millet (*Pennisetum glaucum*) and their possible role in multiple stress tolerance. *Plant Biosystems* **2020**, *154*, 503-514, doi:https://doi.org/10.1080/11263504.2019.1651773.
8. Farahat, M.G.; Mahmoud, M.K.; Youseif, S.H.; Saleh, S.A.; Kamel, Z. Alleviation of salinity stress in wheat by ACC deaminase-producing *Bacillus aryabhattai* EWR29 with multifarious plant growth-promoting attributes. *Plant Archives* **2020**, *20*, 417-429.
9. Baek, D.; Rokibuzzaman, M.; Khan, A.; Kim, M.C.; Park, H.J.; Yun, D.J.; Chung, Y.R. Plant-growth promoting *Bacillus oryzicola* YC7007 modulates stress-response gene expression and provides protection from salt stress. *Front. Plant Sci.* **2020**, *10*, 1646, doi:https://doi.org/10.3389/fpls.2019.01646.
10. Yoolong, S.; Kruasuwan, W.; Pham, H.T.T.; Jaemsaeng, R.; Jantasuriyarat, C.; Thamchaipenet, A. Modulation of salt tolerance in Thai jasmine rice (*Oryza sativa* L. cv. KDML105) by *Streptomyces venezuelae* ATCC 10712 expressing ACC deaminase. *Sci. Rep.* **2019**, *9*, 10, doi:https://doi.org/10.1038/s41598-018-37987-5.
11. Szymanska, S.; Darowska, G.B.; Tyburski, J.; Niedojadlo, K.; Piernik, A.; Hrynkiewicz, K. Boosting the *Brassica napus* L. tolerance to salinity by the halotolerant strain *Pseudomonas stutzeri* ISE12. *Environmental and Experimental Botany* **2019**, *163*, 55-68, doi:https://doi.org/10.1016/j.envexpbot.2019.04.007.
12. Razzaghi Komaresofla, B.; Alikhani, H.A.; Etesami, H.; Khoshkholgh-Sima, N.A. Improved growth and salinity tolerance of the halophyte *Salicornia sp.* by co–inoculation with endophytic and rhizosphere bacteria. *Appl. Soil Ecol.* **2019**, *138*, 160-170, doi:https://doi.org/10.1016/j.apsoil.2019.02.022.
13. Orozco-Mosqueda, M.D.; Duan, J.; DiBernardo, M.; Zetter, E.; Campos-Garcia, J.; Glick, B.R.; Santoyo, G. The production of ACC deaminase and trehalose by the plant growth promoting bacterium *Pseudomonas sp.* UW4 synergistically protect tomato plants against salt stress. *Frontiers in Microbiology* **2019**, *10*, 10, doi:https://doi.org/10.3389/fmicb.2019.01392.
14. Kang, S.M.; Shahzad, R.; Bilal, S.; Khan, A.L.; Park, Y.G.; Lee, K.E.; Asaf, S.; Khan, M.A.; Lee, I.J. Indole-3-acetic-acid and ACC deaminase producing *Leclercia adecarboxylata* MO1 improves *Solanum lycopersicum* L. growth and salinity stress tolerance by endogenous secondary metabolites regulation. *BMC Microbiol.* **2019**, *19*, 14, doi:https://doi.org/10.1186/s12866-019-1450-6.
15. Din, B.U.; Sarfraz, S.; Xia, Y.; Kamran, M.A.; Javed, M.T.; Sultan, T.; Munis, M.F.H.; Chaudhary, H.J. Mechanistic elucidation of germination potential and growth of wheat inoculated with exopolysaccharide and ACC-deaminase producing *Bacillus* strains under induced salinity stress. *Ecotoxicol. Environ. Saf.* **2019**, *183*, 109466, doi:https://doi.org/10.1016/j.ecoenv.2019.109466.
16. Cherif-Silini, H.; Thissera, B.; Bouket, A.C.; Saadaoui, N.; Silini, A.; Eshelli, M.; Alenezi, F.N.; Vallat, A.; Luptakova, L.; Yahiaoui, B., et al. Durum wheat stress tolerance induced by endophyte *Pantoea agglomerans* with genes contributing to plant functions and secondary metabolite arsenal. *Int. J. Mol. Sci.* **2019**, *20*, 36, doi:https://doi.org/10.3390/ijms20163989.
17. Al-Garni, S.M.S.; Khan, M.M.A.; Bahieldin, A. Plant growth-promoting bacteria and silicon fertilizer enhance plant growth and salinity tolerance in *Coriandrum sativum*. *J. Plant Interact.* **2019**, *14*, 386-396, doi:https://doi.org/10.1080/17429145.2019.1641635.
18. Afridi, M.S.; Amna; Sumaira; Mahmood, T.; Salam, A.; Mukhtar, T.; Mehmood, S.; Ali, J.; Khatoon, Z.; Bibi, M., et al. Induction of tolerance to salinity in wheat genotypes by plant growth promoting endophytes: Involvement of ACC deaminase and antioxidant enzymes. *Plant Physiol. Biochem.* **2019**, *139*, 569-577, doi:https://doi.org/10.1016/j.plaphy.2019.03.041.

19. Win, K.T.; Tanaka, F.; Okazaki, K.; Ohwaki, Y. The ACC deaminase expressing endophyte *Pseudomonas spp*. enhances NaCl stress tolerance by reducing stress-related ethylene production, resulting in improved growth, photosynthetic performance, and ionic balance in tomato plants. *Plant Physiol. Biochem.* **2018**, *127*, 599-607, doi:https://doi.org/10.1016/j.plaphy.2018.04.038.
20. Simarmata, R.; Ngadiman; Rohman, S.; Simanjuntak, P. Amelioration of salt tolerance in soybean (*Glycine max.* L) by plant-growth promoting endophytic bacteria produce 1-aminocyclopropane-1-carboxylase deaminase. *Annales Bogorienses-Journal of Tropical General Botany* **2018**, *22*, 81-93, doi:https://doi.org/10.14203/ann.bogor.2018.v22.n2.81-93.
21. Qin, S.; Feng, W.W.; Zhang, Y.J.; Wang, T.T.; Xiong, Y.W.; Xing, K. Diversity of bacterial microbiota of coastal halophyte *Limonium sinense* and amelioration of salinity stress damage by symbiotic plant growth-promoting *Actinobacterium glutamicibacter halophytocola* KLBMP 5180. *Appl. Environ. Microbiol.* **2018**, *84*, doi:https://doi.org/10.1128/aem.01533-18.
22. Chinnaswamy, A.; de la Pena, T.C.; Stoll, A.; Rojo, D.D.; Bravo, J.; Rincon, A.; Lucas, M.M.; Pueyo, J.J. A nodule endophytic *Bacillus megaterium* strain isolated from *Medicago polymorpha* enhances growth, promotes nodulation by *Ensifer medicae* and alleviates salt stress in alfalfa plants. *Ann. App. Biol.* **2018**, *172*, 295-308, doi:https://doi.org/10.1111/aab.12420.
23. Abd Allah, E.F.; Alqarawi, A.A.; Hashem, A.; Radhakrishnan, R.; Al-Huqail, A.A.; Al-Otibi, F.O.N.; Malik, J.A.; Alharbi, R.I.; Egamberdieva, D. Endophytic bacterium *Bacillus subtilis* (BERA 71) improves salt tolerance in chickpea plants by regulating the plant defense mechanisms. *J. Plant Interact.* **2018**, *13*, 37-44, doi:https://doi.org/10.1080/17429145.2017.1414321.
24. Singh, R.P.; Jha, P.; Jha, P.N. Bio-inoculation of plant growth-promoting rhizobacterium *Enterobacter cloacae* ZNP-3 increased resistance against salt and temperature stresses in wheat plant (*Triticum aestivum* L.). *J. Plant Growth Regul.* **2017**, *36*, 783-798, doi:https://doi.org/10.1007/s00344-017-9683-9.
25. Shahzad, R.; Khan, A.L.; Bilal, S.; Waqas, M.; Kang, S.M.; Lee, I.J. Inoculation of abscisic acid-producing endophytic bacteria enhances salinity stress tolerance in *Oryza sativa*. *Environmental and Experimental Botany* **2017**, *136*, 68-77, doi:https://doi.org/10.1016/j.envexpbot.2017.01.010.
26. Qin, S.; Feng, W.W.; Wang, T.T.; Ding, P.; Xing, K.; Jiang, J.H. Plant growth-promoting effect and genomic analysis of the beneficial endophyte *Streptomyces sp.* KLBMP 5084 isolated from halophyte *Limonium sinense*. *Plant and Soil* **2017**, *416*, 117-132, doi:https://doi.org/10.1007/s11104-017-3192-2.
27. Khan, A.L.; Waqas, M.; Asaf, S.; Kamran, M.; Shahzad, R.; Bilal, S.; Khan, M.A.; Kang, S.M.; Kim, Y.H.; Yun, B.W., et al. Plant growth-promoting endophyte *Sphingomonas sp* LK11 alleviates salinity stress in *Solanum pimpinellifolium*. *Environmental and Experimental Botany* **2017**, *133*, 58-69, doi:https://doi.org/10.1016/j.envexpbot.2016.09.009.
28. Irizarry, I.; White, J.F. Application of bacteria from non - cultivated plants to promote growth, alter root architecture and alleviate salt stress of cotton. *Journal of Applied Microbiology* **2017**, *122*, 1110-1120, doi:https://doi.org/10.1111/jam.13414.
29. Ali, A.; Shahzad, R.; Khan, A.L.; Halo, B.A.; Al-Yahyai, R.; Al-Harrasi, A.; Al-Rawahi, A.; Lee, J. Endophytic bacterial diversity of *Avicennia marina* helps to confer resistance against salinity stress in *Solanum lycopersicum*. *J. Plant Interact.* **2017**, *12*, 312-322, doi:https://doi.org/10.1080/17429145.2017.1362051.
30. Yang, A.Z.; Akhtar, S.S.; Iqbal, S.; Amjad, M.; Naveed, M.; Zahir, Z.A.; Jacobsen, S.E. Enhancing salt tolerance in quinoa by halotolerant bacterial inoculation. *Funct. Plant Biol.* **2016**, *43*, 632-642, doi:https://doi.org/10.1071/fp15265.
31. Pereira, S.I.A.; Moreira, H.; Argyras, K.; Castro, P.M.L.; Marques, A.P.G.C. Promotion of sunflower growth under saline water irrigation by the inoculation of beneficial microorganisms. *Appl. Soil Ecol.* **2016**, *105*, 36-47, doi:https://doi.org/10.1016/j.apsoil.2016.03.015.
32. Hashem, A.; Abd Allah, E.F.; Alqarawi, A.A.; Ai-Huqail, A.A.; Wirth, S.; Egamberdieva, D. The interaction between arbuscular mycorrhizal fungi and endophytic bacteria enhances plant growth of *Acacia gerrardii* under salt stress. *Frontiers in Microbiology* **2016**, *7*, 15, doi:https://doi.org/10.3389/fmicb.2016.01089.

33. Egamberdieva, D.; Jabborova, D.; Berg, G. Synergistic interactions between *Bradyrhizobium japonicum* and the endophyte *Stenotrophomonas rhizophila* and their effects on growth, and nodulation of soybean under salt stress. *Plant and Soil* **2016**, *405*, 35-45, doi:https://doi.org/10.1007/s11104-015-2661-8.
34. Cheng, L.; Zhang, N.; Huang, B. Effects of 1-aminocyclopropane-1-carboxylatedeaminase-producing bacteria on perennial ryegrass growth and physiological responses to salinity stress. *Journal of the American Society for Horticultural Science* **2016**, *141*, 233-241, doi:https://doi.org/10.21273/JASHS.141.3.233.
35. Barnawal, D.; Bharti, N.; Tripathi, A.; Pandey, S.S.; Chanotiya, C.S.; Kalra, A. ACC-deaminase-producing endophyte *Brachybacterium paraconglomeratum* strain SMR20 ameliorates Chlorophytum salinity stress via altering phytohormone generation. *J. Plant Growth Regul.* **2016**, *35*, 553-564, doi:https://doi.org/10.1007/s00344-015-9560-3.
36. Singh, R.P.; Jha, P.; Jha, P.N. The plant-growth-promoting bacterium *Klebsiella sp* SBP-8 confers induced systemic tolerance in wheat (*Triticum aestivum*) under salt stress. *J. Plant Physiol.* **2015**, *184*, 57-67, doi:https://doi.org/10.1016/j.jplph.2015.07.002.
37. Gond, S.K.; Torres, M.S.; Bergen, M.S.; Helsel, Z.; White, J.F. Induction of salt tolerance and up-regulation of aquaporin genes in tropical corn by rhizobacterium *Pantoea agglomerans*. *Letters in Applied Microbiology* **2015**, *60*, 392-399, doi:https://doi.org/10.1111/lam.12385.
38. Ali, S.; Charles, T.C.; Glick, B.R. Amelioration of high salinity stress damage by plant growth-promoting bacterial endophytes that contain ACC deaminase. *Plant Physiol. Biochem.* **2014**, *80*, 160-167, doi:https://doi.org/10.1016/j.plaphy.2014.04.003.
39. Qin, S.; Zhang, Y.-J.; Yuan, B.; Xu, P.-Y.; Xing, K.; Wang, J.; Jiang, J.-H. Isolation of ACC deaminase-producing habitat-adapted symbiotic bacteria associated with halophyte *Limonium sinense* (Girard) Kuntze and evaluating their plant growth-promoting activity under salt stress. *Plant and Soil* **2013**, *374*, 753-766, doi:https://doi.org/10.1007/s11104-013-1918-3.
40. Rojas-Tapias, D.; Moreno-Galván, A.; Pardo-Díaz, S.; Obando, M.; Rivera, D.; Bonilla, R. Effect of inoculation with plant growth-promoting bacteria (PGPB) on amelioration of saline stress in maize (*Zea mays*). *Appl. Soil Ecol.* **2012**, *61*, 264-272, doi:https://doi.org/10.1016/j.apsoil.2012.01.006.
41. Cheng, Z.; Woody, O.Z.; McConkey, B.J.; Glick, B.R. Combined effects of the plant growth-promoting bacterium *Pseudomonas putida* UW4 and salinity stress on the *Brassica napus* proteome. *Appl. Soil Ecol.* **2012**, *61*, 255-263, doi:https://doi.org/10.1016/j.apsoil.2011.10.006.
42. Jha, B.; Gontia, I.; Hartmann, A. The roots of the halophyte *Salicornia brachiata* are a source of new halotolerant diazotrophic bacteria with plant growth-promoting potential. *Plant and Soil* **2011**, *356*, 265-277, doi:https://doi.org/10.1007/s11104-011-0877-9.
