# Supplementary material for: Can Bacterial Endophytes Be Used as a Promising Bio-Inoculant for the Mitigation of Salinity Stress in Crop Plants?—A Global Meta-Analysis of the Last Decade (2011–2020)
Source: Microorganisms. 2021 Sep 2;9(9):1861. doi: 10.3390/microorganisms9091861 (PMC8467090; doi:10.3390/microorganisms9091861)
Supplement: Supplementary file 1 [file microorganisms-09-01861-s001.zip › Supplementary Figures_DEF_PoP.pdf]

microorganisms

MDPI

Article

Can Bacterial Endophytes Be Used as a Promising Bio-Inoculant for the Mitigation of Salinity Stress in Crop Plant?—A Global Meta-Analysis of Last Decade (2011–2020)

Muhammad Aammar Tufail[1,2,3], Ana Bejarano[2,3], Awais Shakoor[4], Asif Naeem[5], Muhammad Saleem Arif[6], Afzal Ahmed Dar[7], Taimoor Hassan Farooq[8], Ilaria Pertot[2,3] and Gerardo Puopolo[2,3]

[1] Department of Civil, Environmental and Mechanical Engineering, University of Trento, via Mesiano 77, 38123, Trento, Italy; muhammad.tufail@unitn.it (M.A.T.)
[2] Center Agriculture Food Environment (C3A), University of Trento, via E. Mach 1, 38098, San Michele all'Adige, Italy; ana.bejaranoramos@unitn.it (A.B.), ilaria.pertot@unitn.it (I.P.), gerardo.puopolo@unitn.it (G.P.)
[3] Department of Sustainable Agro-ecosystems and Bioresources, Research and Innovation Centre, Fondazione Edmund Mach, Via E. Mach 1, 38098, San Michele all'Adige, Italy
[4] Department of Environment and Soil Sciences, University of Lleida, Avinguda Alcalde Rovira Roure 191, 25198, Lleida, Spain; awais.shakoor@udl.cat (A.S.)
[5] Institute of Plant Nutrition and Soil Science, Kiel University, Hermann-Rodewald-Strasse 2, 24118, Kiel, Germany; anaeem@plantnutrition.uni-kiel.de (A.N.)
[6] Department of Environmental Sciences & Engineering, Government College University Faisalabad, Faisalabad, 38000, Pakistan; msarif@outlook.com (M.S.A.)
[7] School of Environmental Science and Engineering, Shaanxi University of Science and Technology, 710000, Xian, China; afzaldar@sust.edu.cn (A.A.D)
[8] Bangor College China, a Joint Unit of Bangor University and Central South University of Forestry and Technology, Changsha 410004, China; taimoorhassan2055@gmail.com (T.H.F)

* Correspondence: ana.bejaranoramos@unitn.it

Keywords: plant growth-promoting endophytic bacteria; salinity stress; osmoregulation; antioxidant system; photosynthetic capacity; meta-analysis

Citation: Tufail, M.A.; Bejarano, A.; Shakoor, A.; Naeem, A.; Arif, M.S.; Dar, A.A.; Farooq, T.H.; Pertot, I.; Puopolo, G. Can Bacterial Endophytes Be Used as a Promising Bio-Inoculant for the Mitigation of Salinity Stress in Crop Plants?—A Global Meta-Analysis of the Last Decade (2011–2020). *Microorganisms* **2021**, *9*, 1861. https://doi.org/10.3390/microorganisms9091861

Academic Editor: Gustavo Santoyo

Received: 23 July 2021
Accepted: 26 August 2021
Published: 2 September 2021

Publisher's Note: MDPI stays neutral with regard to jurisdictional claims in published maps and institutional affiliations.

Copyright: © 2021 by the authors. Licensee MDPI, Basel, Switzerland. This article is an open access article distributed under the terms and conditions of the Creative Commons Attribution (CC BY) license (https://creativecommons.org/licenses/by/4.0/).

PRISMA 2009 Flow Diagram

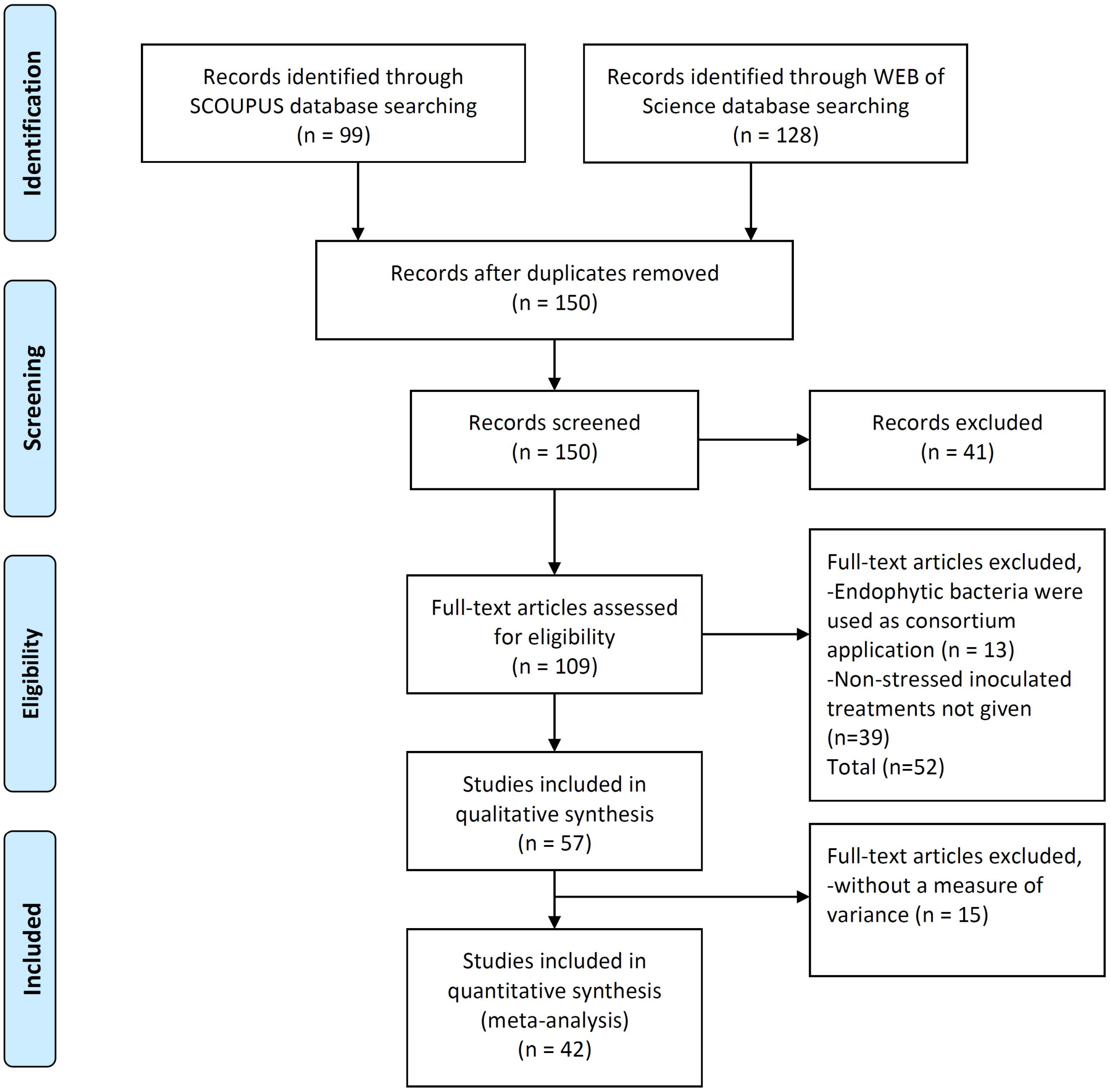

From: Moher D, Liberati A, Tetzlaff J, Altman DG, The PRISMA Group (2009). *P*referred *R*eporting *I*tems for *S*ystematic Reviews and *M*eta-*A*nalyses: The PRISMA Statement. PLoS Med 6(7): e1000097. doi:10.1371/journal.pmed1000097

For more information, visit www.prisma-statement.org.

Figure S1. Preferred reporting items for systematic reviews and meta-analyses (PRISMA) flow diagram for the meta-analysis (Moher et al., 2009, https://doi.org/10.1371/journal.pmed.1000097)

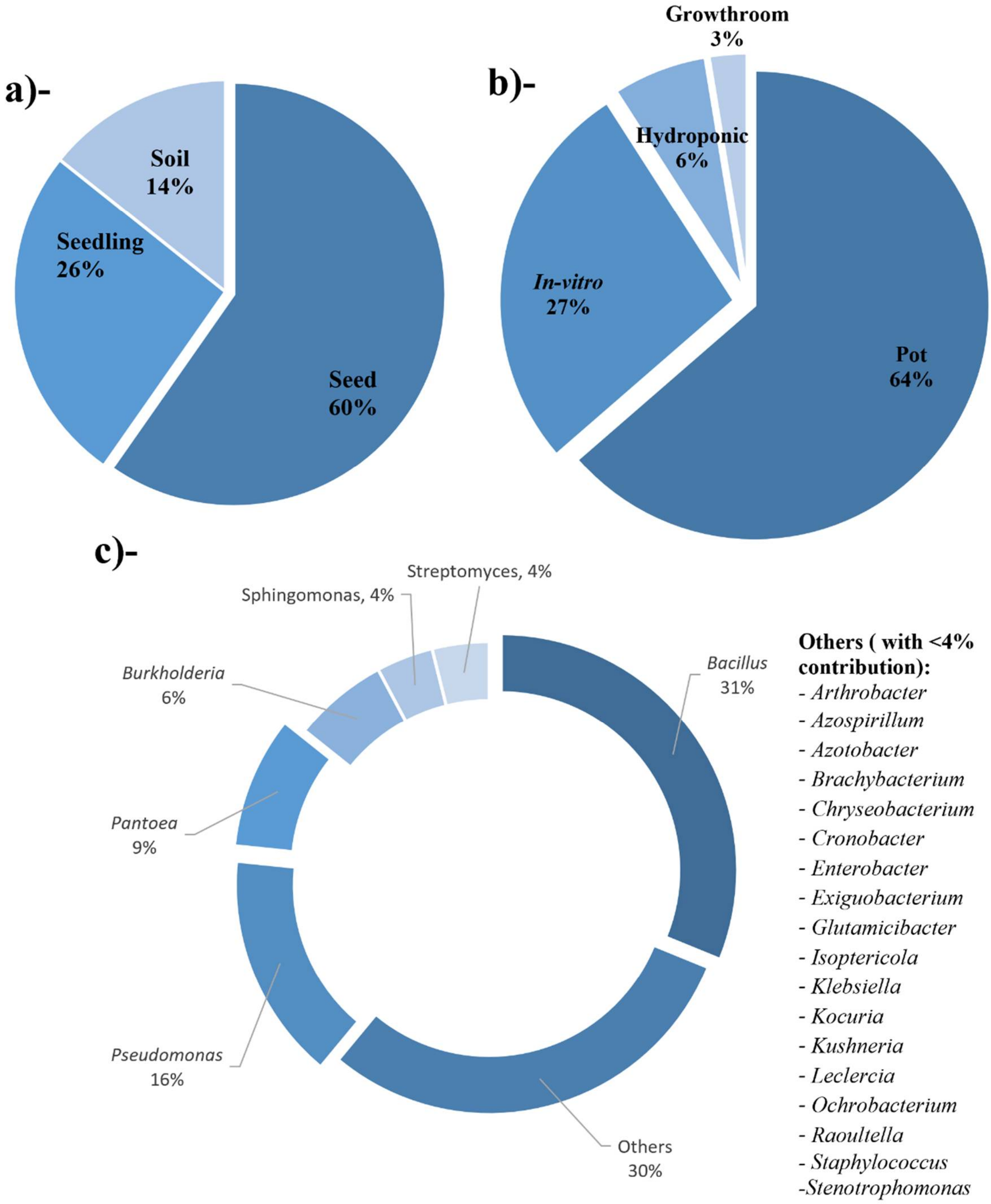

Figure S2. General information about the 1214 observations and 77 experiments obtained from 42 studies used in this meta-analysis, a)- Inoculation method, b)- Experimental conditions, and c)- Genera of bacterial endophytes.
